# The Church and the World

**Published:** 1879-02

**Authors:** 


// HALL'S JOURNAL OF HEALTH.

WE AIM TO SHOW HOW DISEASE MAY BE AVOIDED, AND THAT IT IS BEST, WHEN SICKNESS COMES, TO TAKE NO MEDICINE WITHOUT CONSULTING A PHYSICIAN.

FEBRUARY, 1879

### THE CHURCH AND THE WORLD.

There never was a time when Christianity flourished more, spread faster, extended farther, than when the line of demarkation between the church and the world was most clear and distinct; than when to be a Christian was to be despised, persecuted, killed. In those days, men were not ashamed of their religion; for, if they were religious at all, their piety was of so sterling a stamp, that it was unconcealable; it shone like the sun, it was seen in every act, it was heard in every word, it was felt in every presence; there was that about a man, which, without a word or act, said, "He is a Christian." On the other hand, in proportion as the great line is indistinct; in proportion as there is an amalgamation between the church and the world, a commingling of views and policies and practices; in proportion as the spirit of accommodation prevails; in such proportion, is piety weak, watery, empty. As observant men grow old, they who are not specially under the influence of religious principle, their motto is, more and more, "policy." For this, they suppress personal independence, suppress opinion, suppress conscience. It is the same with communities and nations. Thus, an unchristianized civilization is pretence without reality, is form, is ceremony, is a grand sham; words are used to conceal ideas, and Talleyrandism is the order of the day.

The price of liberty is eternal vigilance, and by no other means can a sound practical Christianity prevail in any family or community or nation. Hence, it behooves the best friends of religion to exercise a ceaseless vigilance against the commingling of sentiments and practices, as between the church and the world.

In a heroic independence, the church is found largely wanting, in the present age; and, by an assumption of the principle, that they who are in Rome must do as Rome does, there is no distinction, in a general way, between the dress of the church and that of the world. Our wives and daughters do not "adorn themselves in modest apparel," but "with embroidered hair and gold and pearls and costly array;" they greedily catch up the extravagances of hollow courts abroad; and the question as to dress, is not whether it is appropriate, but, "Is it fashionable? Does it prevail in foreign courts?" And, up to this time, there is no absurdity or indecency of dress practiced at the Court of the Tuileries, that is not forthwith adopted here; and the habiliments at the altar of devotion are the same as those worn in the *salons* of royalty. This subject is antiquated and threadbare, and is dismissed with the single suggestion, that the true and old nobility of England is distinguished by its plain and convenient and useful attire, leaving it to the servants of the household to glory in extremest fashion. The children of the great King would do well to take a lesson therefrom, and, at least, let the medium of fashion be their guide.

Truthfulness is a defect among Christians. The most expressive adjectives, the fullest expletives, the strongest forms of speech, are brought into constant requisition, in the most trivial affairs of life. A little dust, an inconvenient wind, a slight shower, makes the weather "horrid." The muttering of distant thunder, is "awful;" and "outrageous," and "disgusting," and "too contemptible for anything," leap to the lips of our daughters, with the facility of the most endearing expressions. "I am pleased to meet with you," is too tame a phrase in case of an ordinary introduction; "I am extremely happy to make your acquaintance," is the uniform utterance. Half the letters begin and end with a mechanical lie, instead of a conscientious gauge of phrase. If all are treated thus alike, where is the encouragement to virtue? where is the wholesome frowning on vice? The true rule should be, meet the humble, working Christian, whether in rags or ermine, with the cordiality and equality of a brother; but meet the *roué*, the gambler, the defrauder, the Sabbath-breaker, and the outlaw, in whatever direction that outlawry may exhibit itself, with a dignified distance, yet warmed with compassion. In shorter phrase, make virtue feel that it is encouraged, and vice that it is frowned upon. Let justice and truth be exhibited in every act of life.

Gambling is steadily and stealthily, under false guises, creeping into the church. "Let us do evil that good may come," is a maxim as false as it is mischievous. A past generation had no difficulty in instituting lotteries for purposes of benevolence. Scattering attempts to do the same now meet with a general and stern rebuke. Still, it is not an unknown thing, at this present writing; although every State in the Union, which has any self-respect, has banished the lottery from the statute-book, taking in reality the lead of the church.

That is essentially a gambling transaction of the baldest kind, where there is a possible benefit, on a given occurrence, bearing no adequate proportion to what is hazarded, and which is not dependent on one's own efforts; that is, where a man has no power, honestly, to bring about that given occurrence.

All gift transactions come within this rule. So does the purchase of a slice of cake, at a church fair, for a penny, when some one is to get a slice which has within it a gold ring, or other valuable commodity.

"Life Insurance," more properly, in a moral sense, called "Life Assurance," in England, is of this form of gambling. One church has a kind of an official organization of this sort for the benefit of its ministers. These establishments pay large salaries, and large dividends, showing that they are largely profitable, and just in that proportion the benefit is all on one side. The commandment of the Church is, "Leave thy fatherless children; I will preserve them alive; and let thy widows trust in Me." It is as applicable now as it was in Jeremiah's day, and has not lost its meaning. If a man works diligently and does right, in the fear of God, as large a share of worldly prosperity will be allowed to him and his, as is considered by the Almighty to be safe and good; and any invocation of mere chance, which will act as an interference with this order of things, or which will be in the nature of a counteracting agency, is not allowable.

Any profit derived from a common life insurance institution is, in a measure, practically, the price of blood, for death only brings advantage. And more, the dividends and premiums are paid mainly from the hard earnings of the poor and the unfortunate, as it is chiefly the poor who seek the benefits of these establishments, mechanics, clergymen, and salaried persons, who have pretty much given up the idea of being rich, or even comfortably forehanded. It is they who barely make a living, who are the chief patrons of life insurance; persons who pay the premiums with effort, and, sometimes, with painful self-denials, with harassing turn rounds, and, occasionally, with ruinous sacrifices; for, if the premiums are not paid at a certain hour, there is the forfeiture of all that has been paid for years, for a lifetime; and it is from those whom such a forfeiture fails to stimulate to payment, that these companies derive a large share of their profits. A worthy clergyman, or a poor mechanic, has been paying his premiums punctually for years, but sickness, or accident, or financial pressure, which none could foresee or prevent, make it an almost impossible thing to meet the premium—the very sickness, perhaps, which is to remove him from the world! Can any one measure the desperation of effort under such circumstances, to secure the needed amount? The very apprehension of inability to do so, may be enough, sometimes, to press a man into the grave, who, but for that, might have risen above his disease. All insurance companies of this kind, of lengthened experience, know that large profits arise from lapsed policies, policies forfeited by some legal quibble, by forgetfulness, by absolute inability to continue the premium.

No intelligent man will deny these statements, hence the proof will not be entered into.

These views are modified where there is a just reciprocity of interest, as in the case of those organizations founded on the "Mutual" plan. As to the justness or morality of insurances on personal and real estate, nothing is here said; that must take care of itself. The question under discussion now is, as to simple life assurance, whether a Christian man is not amalgamating with the worldling in patronizing these institutions? Otherwise, where is there any distinctive trust in God? In what regard does the churchman and the worldling differ? Surely, in this thing there is an abnegation of the express command, "Come out from among them, and be ye separate." This is clear. It is imperative. It comes with a "Thus saith the Lord."

A truer policy, against which there can be no great objection, is, to take the same amount of premium which would be paid to a company, and put it out on bond and mortgage with a wise care; then the loss of it becomes, to a great extent, an impossibility; equal in safety, perhaps, is an investment in the funds of the general government, and, next to that, in savings' banks. By thus investing, and taking up the interest quarterly or semi-annually, a man's family will, if he begin at twenty-five, or on the day of his marriage, have, at the age of fifty years, about double the amount that he would have had, had he invested in a life insurance, and died at that age, and have it, too, with a certainty. No contingencies to harass his last hours, as to the solvency of the company, as to quibbling evasions, as in a recent case, where a life insurance company repudiated the payment of a policy paid faithfully and regularly by a woman, for years and years in succession, on the ground that it was nothing but a bet, and that the law did not enforce such claims; and yet the directorship of that company had among it the honored names of some of our merchant princes.

If it be urged, that men would not faithfully pay up and reinvest, on the plan of a loan, the reply is, the fault is not in the principle, but in the man. Because, it avails more with some to sign a temperance pledge, than would a verbal promise or simple resolution, that does not detract from the superior merit of abstaining, from stern, unaided principle, rather than from a formal or mechanical compulsion. It is the motive of the abstinence which gives it high character. He is the grandest man, who acts from principle alone. He is pitiful, who does right from compulsion.

